# Paracetamol *versus *placebo in treatment of non-severe malaria in children in Guinea-Bissau: a randomized controlled trial

**DOI:** 10.1186/1475-2875-10-148

**Published:** 2011-06-01

**Authors:** Poul-Erik Kofoed, Johan Ursing, Amabelia Rodrigues, Lars Rombo

**Affiliations:** 1 Projecto de Saúde de Bandim, Apartado 861, 1004 Bissau Codex, Guinea-Bissau; 2 Health Services Research Unit, Lillebaelt Hospital/IRS, University of Southern Denmark, Kolding Hospital, 6000 Kolding, Denmark; 3 Department of Medicine, Unit of Infectious Diseases, Karolinska University Hospital, Solna, Sweden; 4 Centre for Clinical Research, Sormland County Council, Kungsgatan 41, 631 88 Eskilstuna, Sweden

## Abstract

**Background:**

The current guidelines for treatment of malaria include paracetamol to children with fever. No convincing evidence for the beneficial effects of this practice exists. Studies show that time to parasite clearance is significantly longer in children treated with paracetamol, which questions the policy. Whether this is of clinical importance has not been investigated.

**Methods:**

Children with *Plasmodium falciparum *monoinfection and ≥20 parasites per 200 leucocytes at the Bandim Health Centre, Guinea-Bissau were randomized to receive paracetamol or placebo together with chloroquine for three days in a double blind randomized study. Temperature and symptoms were recorded twice daily during treatment and on day 3. The participants were interviewed and a malaria film taken once weekly until day 35. The data is in the form of grouped failure-times, the outcome of interest being time until parasitaemia during follow-up. Mantel-Haenszel weighted odds ratios are given. Other differences between and within the two groups have been tested using the Chi-square test and Mann-Whitney U test.

**Results:**

In the evening of the day of inclusion, the temperature was slightly, but statistically insignificant, higher in the placebo group and significantly more children complained of headache. At no other time was a significant difference in temperature or symptoms detected. However, 6 children from the placebo-group as compared to two children from the paracetamol-group were admitted to hospital with high fever and convulsions by day 3. No differences in the cumulative percentages of children with adequate clinical and parasitological response were found in the intention-to-treat analysis or in the per-protocol analysis.

**Conclusion:**

Fewer children had early treatment failure and the mean temperature was slightly lower in the afternoon on day 0 in the paracetamol group. However, the cumulative adequate clinical and parasitological cure rates were not significantly different during the period of study. It is doubtful whether adding paracetamol to the treatment of uncomplicated malaria in children is beneficial.

**Trial registration:**

NCT00137566.

## Background

Fever is a common symptom of many childhood illnesses. Although the disease process may be harmful, there is no evidence that fever is harmful in itself. In fact, fever may be beneficial by enhancing the host response to infection [1,2]. There are few prospective human studies on whether antipyretics have any clinically relevant effects. According to a Cochrane Review, evidence that paracetamol has a superior antipyretic effect compared with placebo is inconclusive [3]. Still, many parents and physicians believe that antipyretic treatment improves the comfort of febrile children and antipyretics are therefore commonly prescribed. Although paracetamol is generally regarded as a safe antipyretic drug, liver failure is a well-known consequence of paracetamol overdose [4,5] and multiple doses of paracetamol, only marginally greater than the recommended maximum dose, might cause liver damage [6], especially in febrile and acutely malnourished children [7].

The role of fever in malaria is unclear. Tumour necrosis factor (TNF) is an important mediator of fever in malaria [8,9], and experimental data suggest that both TNF and fever have anti-parasitic properties [10,11]. In a small study, Krishna *et al *found a longer parasite clearance time when paracetamol was used [12]. This finding was confirmed by a study in Gabon in which children with non-severe *P. falciparum *malaria were randomized to receive mechanical antipyretics (continuous fanning, tepid sponging and cool blankets) either alone or in combination with paracetamol [13]. Time to parasitic clearance was significantly longer and the level of TNF reduced in the paracetamol group suggesting that the longer parasite clearance time could be due to decreased production of TNF and oxygen radicals [13].

The current WHO guidelines on the management of fever recommend the use of paracetamol for children with a temperature of 38.5°C or above [14] and the National Malaria Programme in Guinea-Bissau recommends paracetamol for all children treated for malaria. Still, the costs are not negligible and the adverse events can be serious [4-7,15]. It is therefore important to evaluate if treatment with paracetamol for non-severe malaria is beneficial for the child [4].

## Methods

### Procedures and patients

The study was performed in the area of Bandim on the outskirts of Bissau, Guinea-Bissau. Parents from Bandim attending the Bandim Health Centre with children weighting more than 7.5 kg and having fever or other symptoms compatible with malaria and stating that the children had not taken any antimalarial drug during the previous week were informed of the study. Included children had a thick film examined for malaria. Children with convulsions, severe vomiting, severe anaemia, a severe concurrent infection or who for other reasons were considered in need of hospital care were not eligible.

Children with mono-infection with *P. falciparum *and 20 or more parasites per 200 leukocytes (= 800/μl assuming a leukocyte count of 8000/μl) were enrolled from June 2004 to July 2006. Children were allocated to one of six 5 kg-interval weight groups or to a group of children with a body-weight above 37.5 kg. For each weight group, numbered boxes had been prepared at random with either paracetamol tablets or undistinguishable placebo tablets corresponding to approximately 50 mg paracetamol per kg bodyweight per day for three days. The children were given study numbers consecutively within each weight-group. The first dose of paracetamol/placebo from the tablet-box corresponding to the study number of the child was given by an experienced nurse at the health centre. For logistic reasons (home-visits could not be performed at intervals matching the intervals between medications) the mothers were then given the tablet-box containing tablets until the end of day 2 and were carefully instructed on how to give the tablets. A health worker visited the house on day 3 and asked to see the tablet-box and counted the number of remaining tablets. The randomisation numbers were kept separately at the Department of Paediatrics in Kolding, Denmark. An interim analysis was made by one of the researchers not involved in the recruitment of the patients when a total of 40 and 140 children had completed the follow-up to ensure that none of the groups had an unacceptable high rate of recurrent parasitaemia using the results of previous in-vivo chloroquine studies as guidelines [16-18].

Following recommendations of the National Malaria Programme at the time of the study, 25 mg/kg bodyweight of chloroquine was split into daily doses of 10, 10 and 5 mg/kg and given days 0, 1 and 2 for treatment of malaria. An experienced nurse ascertained that the tablets were swallowed and recorded the time the dose was given. If the child did not turn up as planned, a project health worker went to the house to ensure the medication. If the child vomited within 30 minutes after receiving the tablets, the dose was repeated. If the child vomited again during the next 30 minutes, a dose of 10 mg Quinimax^® ^dihydrochloride (Sanofi Winthrop, Gentilly, France) per kg bodyweight was given intramuscularly and the child was referred to hospital.

Tablets with 160 mg chloroquine-phosphate (= 100 mg chloroquine base) were obtained from Recip SB, 12054 Årsta, Sweden. Paracetamol tablets of 500 mg and undistinguishable placebo tablets were obtained from GlaxoSmithKline, Dungarvan, Irland.

The children or their mothers were asked for any symptoms twice daily on day 0, day 1 and day 2 and in the evening on day 3. At the same time, the axillary temperature was measured using an electronic thermometer.

### Follow-up

Following treatment, the children were visited once weekly until day 35 for thick and thin films. The mothers were interviewed about any medication given since the last visit, and the health worker assessed the condition of the child. During the study period, the parents were asked to bring the child to the health centre in case of any illness to ensure early detection of clinical malaria. All treatments were free during the study-period. Recrudescent infections were treated according to the national recommendations which were then sulphadoxine/pyrimethamine or, for severe cases, quinine.

### Microscopy

Before inclusion, a thick and a thin film were examined. Thick films were also examined on day 3, 7, 14, 21, 28 and 35 after initiation of the treatment and whenever a child sought medical attention at the Bandim Health Centre due to fever or other symptoms suggestive of malaria. To ensure the quality of the microscopy, 10% of the thick films were re-examined by an experienced laboratory technician. The parasites were counted per 200 leukocytes. If abundant, the number of leukocytes was counted per 500 parasites.

### Polymerase chain reaction

Each time a thick smear was made, approximately 100 μL of blood was also put onto filter paper, dried and then stored in a separate sealed plastic bag. Approximately 50 μl of blood was cut from the filter papers and DNA was extracted using an ABI Prism 6100 Nucleic Acid Prep Station (Applied Biosystems, Fresno, CA) according to the manufacturer's recommendations. Extracted DNA was frozen at -20°C until amplification by PCR. Merozoite surface protein 1 (*pfmsp1*) and merozoite surface protein 2 (*pfmsp2*) were amplified by PCR as described previously [19]. PCR products were separated on agarose gels (Amresco, Solon, OH), stained with ethidium bromide and visualised under UV trans-illumination (BioRad GelDoc System, BioRad, Hercules, CA). *Pfmsp1 *and *pfmsp2 *were amplified from DNA collected on the day of inclusion and the day of failure. Recrudescent infections were defined as reappearance of at least one band from both *pfmsp1 *and *pfmsp2 *during the follow-up or the re-appearance of a band in the successfully amplified gene if either *pfmsp1 *or 2 failed. Re-infections were defined as those where no band re-occurred. Whenever a child received antimalarial medication elsewhere, the blood sample drawn on the last visit was analysed.

### Outcome measures

Early treatment failure (ETF) was defined as 1) development of severe malaria or appearance of danger signs on day 1 to 3; 2) positive malaria film and temperature ≥ 37.5°C on day 3; or 3) parasitaemia on day 3 ≥ 25% of the parasitaemia on day 0. Late clinical failure (LCF) was defined as either; 1) fever and a positive malaria film; 2) a history of fever and clinical symptoms of malaria as well as a positive malaria film. Late parasitological failure (LPF) was defined as reappearing parasiteamia on day 7 or later for children without ETF or LCF. Adequate clinical and parasitological response (ACPR) was defined as absence of parasitaemia without the child meeting any of the criteria of ETF, LCF or LPF.

The primary outcome was the cumulative PCR-uncorrected ACPR rate until day 35 and PCR-corrected failure rates on day 28 and 35. Secondary outcomes were the ETF rates, the symptoms as reported by the mothers, the temperature measured during the treatment with paracetamol/placebo; and possible clinical adverse effects of the medication given.

### Statistical analysis

An intention-to-treat analysis was performed to answer the question as to whether the treatments were valuable for the children [20,21]. Therefore, all children admitted to hospital on the day of inclusion were considered as ETF, and all children treated by a third person with an anti-malarial during follow-up were considered as LCF. Children who could not be found during medication or the first follow-up visit due to unknown addresses were considered to have violated the entry criteria of living in Bandim and were therefore excluded.

A per protocol analysis was done to evaluate the efficacy of the treatments. Children admitted to hospital on the day of inclusion were then excluded as they were considered to be so sick that they had been included in violation of the inclusion criteria. Children treated outside the study during follow up without having re-parasitaemia confirmed by a positive malaria film and/or a positive PCR-analysis were considered withdrawal of consent and excluded on the day of retreatment.

The data is in the form of grouped failure-times, the outcome of interest being time until parasitaemia during follow-up. Mantel-Haenszel weighted odds ratios are given. Loss to follow-up between two analysis times has been treated by censoring at the beginning of the time interval. Other differences between and within the two groups have been tested using the Chi-square test and Mann-Whitney U test.

### Ethics

The protocol stated that any study group should be terminated if parasitaemia reappeared in 50% or more of at least 40 children. Ethical clearance was obtained from Direcção de Higiene e Epidemiologia, Ministério da Saúde Publica in Guinea-Bissau (030/DHE/2004) and the Central Ethical Committee in Denmark (2004-7041-11). Children and/or their parents were informed of the study orally as the literacy rate is low. Written information in Creole was given on request. The information was standardised and in accordance with the principles of the Helsinki declaration. This trial was registered at ClinicalTrials.gov [22] with the study ID: PSB-2004-paracetamol (NCT00137566).

## Results

**Figure 1:**
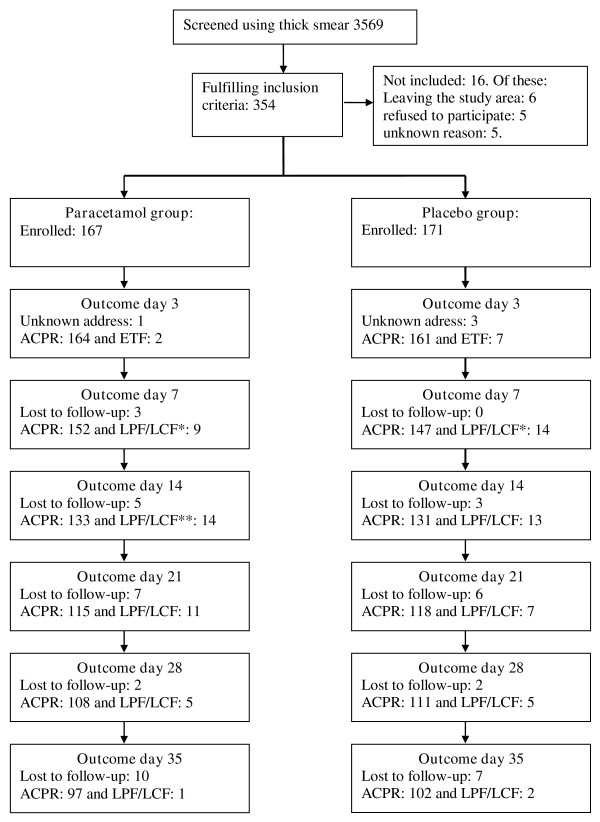
**The reasons for lost to follow-up were mainly due to the children travelling outside the study-area (20 and 16 in paracetamol and placebo groups, respectively)**. One child in the paracetamol group withdrew consent on day 14 and for one child in each of the study groups no information could be obtained. Due to the staff being involved in the national vaccination campaign 2 children were lost to follow-up on day 28 and 3 on day 35. LPF/LCF: including children treated outside the study. *: Including one child from each of the groups admitted to hospital. **: Including one child in the paracetamol group admitted to hospital.

A total of 14.861 children were screened at the Bandim Health Centre and 3.569 had a malaria film examined due to symptoms compatible with malaria. Of these, 354 had 20 or more parasites per 200 leucocytes. Six children were not included as they were leaving the study area and 5 were not included for unknown reasons. Five children or their caretakers refused to participate. The remaining 338 children who fulfilled the inclusion criteria were randomised into one of the two study groups. The number of children lost to follow-up and the number of children with reappearing parasitaemia are shown in Figure 1. There were no differences in sex, age, body-weight, dose of chloroquine received per kg body-weight or parasite density on inclusion between the two groups (Table 1).

**Table 1:** Description of the children included

Treatment group	Paracetamol	Placebo
Males/females	91/76	85/86

Age (months)^a^	62 (3 - 169)	61 (8 - 190)

Weight (kg)^a^	16 (8 - 48)	15 (8 - 43)

Dosage of chloroquine^a,b^	27 (20 - 36)	27 (15 - 33)

Parasitaemia on inclusion^c^	554 (130 - 1186)	643 (191 - 1132)

a) Median (range in brackets)

b) Mg per kg bodyweight

c) Number of parasites per 200 leucocytes. Median with 25% - 75% percentiles

In the paracetamol and the placebo groups 59% (99/167) and 59% (100/170) of the children, respectively claimed to have taken paracetamol during the past 12 hours prior to inclusion. During the three days of treatment, only 1 (0.3%), 14 (4.1%) and 17 (5.0%) children received paracetamol or other anti-pyretic apart from what had been prescribed on inclusion. On day 3, after ending the treatment prescribed at the health centre, a total of 34 children (10.1%) were given paracetamol. There were no differences between the two study groups on any of the days.

When asked to show the tablet-box on day 3, 39% (62/161) and 37% (60/161) in the paracetamol and the placebo groups respectively, still had tablets left with a median number of 1.5 (range 0.5 - 6.0) vs. 2.0 (range: 0.5 - 6.0). The rest showed empty boxes. Assuming that the children had taken the tablets removed from the boxes, the median dose during the 3 days were 136 mg paracetamol/kg (range: 0 - 188) and 143 mg placebo/kg (range: 0 - 188), respectively (p = 0.34).

On inclusion, there were no differences between the groups in the symptoms and signs reported by the mothers. The most frequent symptoms and signs were that the child felt/looked ill (99%, 334/338), had fever (87%, 295/338), had eaten less (84%, 285/338), had headache (83%, 279/338), vomited (41%, 138/338), had diarrhoea (14%. 49/338), and drank less (7%, 22/338). In the evening of day 0, more children in the placebo group complained of headache than in the paracetamol group (105/164 vs. 86/165, p = 0.03). For all other complaints, no statistical significant differences were found during the first three days (data not shown). On day one, three children in the paracetamol group and eight children in the placebo group complained of itching (p = 0.13), but continued the anti malarial treatment. Due to vomiting, 11 children in the paracetamol group and two children in the placebo group had the first dose of chloroquine repeated. The second dose was repeated in four and three children respectively. On day 3, parasites were detected in 19 and 21 of the children in the paracetamol and the placebo group, respectively (OR: 0.88 (95% confidence interval: 0.43 - 1.79)).

The temperature was measured in the morning and evening of day 0, day 1 and day 2 and in the evening on day 3 (Table 2). In the evening of day 0, the temperature was higher in the placebo group than in the paracetamol group (p = 0.01), however when applying the Bonferroni's correction for multiple comparisons this finding was not statistically significant (p should be less than 0.007 for detecting a statistically significant difference at the 5% level). The six children admitted to hospital due to high fever and convulsions during the three days of treatment had a median temperature of 38.5°C on inclusion (37.4°C - 39.9°C) as compared to 37.6°C (36.7°C - 38.9°C) for the children not admitted to hospital during the treatment (p = 0.27).

**Table 2:** Temperature of the children measured in the axil (median with 25% and 75% percentiles in brackets)

	Paracetamol group	Placebo group	
On inclusion	37.6 (36.7 - 39.0)	37.8 (36.7 - 38.9)	P = 0.6

Day 0 evening	36.8 (36.2 - 37.6)	37.2 (36.6 - 38.3)	P = 0.01

Day 1 morning	36.5 (36.0 - 36.9)	36.4 (36.0 - 36.9)	P = 0.8

Day 1 evening	36.1 (35.7 - 36.7)	36.2 (35.8 - 36.7)	P = 0.4

Day 2 morning	36.4 (36.0 - 36.7)	36.3 (35.8 - 36.7)	P = 0.2

Day 2 evening	35.9 (35.5 - 36.3)	36.0 (35.6 - 36.3)	P = 0.3

Day 3 evening	35.9 (35.5 - 36.2)	35.9 (35.4 - 36.1)	P = 0.5

A significant difference in temperature was only found in the evening of the day of inclusion (Mann-Whitney test).

One child from the paracetamol group was admitted to hospital on each of the days 2 and 3. From the placebo group, 3, 2 and 1 children were admitted on day 0, day 1, and day 3, respectively. All were admitted due to high fever and convulsions (2/167 vs. 6/171. OR: 0.33 (0.03 - 1.90)). Two children in the paracetamol group were admitted to hospital after the end of the treatment, one at day 7 and one at day 14, both due to high fever. One child in the placebo group was admitted at day 5 due to anaemia.

A total of 72 children had paired blood-samples from day 0 and the day of reappearing parasitaemia analysed. Filter-paper-samples from six children with LPF or LCF were not available on the day of reparasitaemia, including samples from three children admitted to hospital after end of treatment and 2 treated outside the study. *Pfmsp *1 and 2 amplification failed in 11 samples collected on the day of treatment failure, three of these were treated outside the study. No differences in the cumulative percentages of children with ACPR were found, in the intention-to-treat analysis or in the per-protocol analysis (Tables 3 and 4).

**Table 3:** Intention to treat analysis of the effect of treatment with chloroquine 25 mg/kg

	Paracetamol-group*	Placebo-group*	OR with confidence interval**
Day 0	100% (167/167)	100% (171/171)	

Day 7	93.3% (152/163)	87.5% (147/168)	0.51 (0.21 - 1.15)

Day 14	84.4% (133/147)	79.6% (131/144)	0.72 (0.40 - 1.28)

Day 21	77.0% (115/126)	75.1% (118/125)	0.87 (0.52 - 1.43)

Day 28	73.6% (108/113)	71.9% (111/116)	0.89 (0.56 - 1.41)

Day 35	72.8% (97/98)	70.5% (102/104)	0.87 (0.55 - 1.37)

The adequate clinical and parasitological response (ACPR) rates in the two treatment groups (see text):

*) Cumulative percentages of children with ACPR during follow-up. The number of children with ACPR/total number of children is given in parenthesis (on day 7 children admitted to hospital on day 0 and children with early treatment failure are included in the denominator).

**) Cumulative odds ratio of having ACPR in the group treated with placebo in relation to the paracetamol-group (Mantel-Haenszel weighted odds ratio, 95% confidence interval in brackets).

**Table 4:** Per protocol analysis of the effect of treatment with chloroquine 25 mg/kg

	Paracetamol-group*	Placebo-group*	OR with confidence interval**
Day 0	100% (167/167)	100% (171/171)	

Day 7	93.3% (152/163)	89.1% (147/165)	0.57 (0.24 - 1.33)

Day 14	84.4% (133/147)	81.0% (131/144)	0.75 (0.41 - 1.35)

Day 21	78.2% (115/124)	76.5% (118/124)	0.85 (0.51 - 1.42)

Day 28	76.8% (108/110)	73.2% (111/116)	0.80 (0.49 - 1.30)

Day 35	76.0% (97/98)	72.5% (102/103)	0.80 (0.49 - 1.29)

The adequate clinical and parasitological response (ACPR) rates in the two treatment groups (see text):

Excluded due to violation of the protocol: In the paracetamol group 2 and 3 children were treated

outside the study on day 21 and day 28, respectively. In the placebo-group 3 children were admitted to hospital on the day of inclusion and 1 child was treated outside the study on each of the days 21 and 35.

*) Cumulative percentages of children with ACPR during follow-up. The number of children with ACPR/total number of children is given in parenthesis (on day 7, children with early treatment failure are included in the denominator).

**) Cumulative odds ratio of having ACPR in the group treated with placebo in relation to the paracetamol-group (Mantel-Haenszel weighted odds ratio, 95% confidence interval in brackets).

There were 25 and 16 PCR adjusted LPF + LCF in the paracetamol and placebo groups respectively (OR: 1.55 (0.78 - 3.16)) (Table 5). When added to the 2 and 7 ETF a total of 27 and 23 treatment failures were found (OR: 1.16 (0.63 - 2.15)) (Table 5). The cumulative OR of having ETF or PCR adjusted LPF or LCF on day 7 or before vs. after day 7 was 0.49 (0.18 - 1.24) and 2.72 (1.07 - 7.77), respectively.

**Table 5:** Cumulative percentages of children with recrudescent infections during follow-up in the two treatment groups

	Paracetamol	Placebo	OR with confidence interval*	OR with confidence interval**
ETF	1.2% (2/167)	4.1% (7/171)	0.28 (0.03 - 1.53)	

Day 4 - 7	4.3% (6/161)	9.5% (9/161)	0.49 (0.18 - 1.24)	0.88 (0.25 - 2.86)

Day 8 - 14	12.2% (12/144)	12.1% (4/135)	0.98 (0.49 - 1.95)	1.36 (0.62 - 3.08)

Day 15 -21	16.6% (6/121)	14.3% (3/120)	1.12 (0.60 - 2.07)	1.49 (0.74 - 3.04)

Day 22 - 28	17.4% (1/109)	14.3% (0/111)	1.16 (0.63 - 2.15)	1.55 (0.78 - 3.16)

Day 29 - 35	17.4% (0/97)	14.3% (0/103)	1.16 (0.63 - 2.15)	1.55 (0.78 - 3.16)

Cumulative percentages of children with recrudescent infections during follow-up. The number of children with recrudescent infections/total number of children is given in parenthesis.

*) The OR (with confidence intervals in brackets) of having recrudescent infections in the paracetamol group as compared to the placebo group when recrudescent infections are defined as PCR-proven recrudescent infections plus ETF.

**) The OR (with confidence intervals in brackets) of having recrudescent infections in the paracetamol group as compared to the placebo group when recrudescent infections are defined as PCR-proven recrudescent infections.

## Discussion

Common to other febrile diseases, children suffering from clinical malaria are mostly treated with paracetamol in addition to an anti-malarial drug. Some animal studies have shown that fever increases survival during infection whilst antipyretics increase mortality [23-26]. As parasites exposed to paracetamol were less likely to be eliminated, concerns about the routine use of paracetamol have been raised [12,13].

In the present study we compared the outcome when children with uncomplicated malaria were treated with chloroquine, the anti-malarial recommended by the National Malaria Control Programme in Guinea-Bissau at the time, plus either placebo or paracetamol. The fact that parents consider paracetamol important was confirmed as approximately 60% of the children had received paracetamol prior to inclusion and that most of the paracetamol/placebo prescribed was taken during the 3 days of treatment.

Almost all children suffered from symptoms generally believed to be alleviated by paracetamol like feeling ill, eating less, and having a headache. In the afternoon on the day of inclusion, a higher proportion of children in the placebo group complained of headache. Otherwise, no differences in symptoms were reported by the mothers. This corroborates the findings in the studies cited in a Cochrane review where no differences in resolution of symptoms were found between febrile children treated with paracetamol or placebo/physical methods [3]. Similarly, in a review on antipyretic measures for treatment of fever in malaria, one study reported that paracetamol alleviated headache whereas no other differences in the frequency of symptoms were found in the other studies cited [27].

Vomiting is a major problem in children with malaria as it interferes with the absorption of anti-malarials. A study suggested that febrile patients were more likely to vomit when treated with mefloquine, concluding that antipyretics might improve the management of malaria [28]. However, there was no reduction on the incidence of early vomiting neither with paracetamol nor with tepid sponging in another study [29]. In the present study, no differences were found between the groups in the number of children complaining of vomiting or in the number having the second dose of chloroquine repeated.

The authors of the Cochrane review who evaluated the effect of paracetamol for treatment of fever in children concluded that only "inconsistent and weak evidence supports the use of paracetamol to reduce fever in children" as "the number of reliable studies" are too few [3]. In the present study, a lower temperature was found in the afternoon on day 0 in the paracetamol group. However, applying Bonferroni's correction for multiple comparisons this result is not statistically significant.

Many clinicians treat fever with paracetamol to prevent febrile convulsions but there is little evidence showing that antipyretic treatment reduces the risk of febrile convulsions [4,30]. However, in accordance with the improved fever clearance, only two children from the paracetamol group were admitted to hospital due to high fever and convulsions on day 0 to day 3 as compared to 6 children from the placebo group, indicating that paracetamol might play a role in reducing the number of children suffering from convulsions.

The number of children with parasitaemia on day 3 was identical in the two treatment groups and no difference in the number of children with PCR-adjusted treatment failures was found, but three times as many children in the paracetamol group were affected when looking on failures after day 7. However, this higher risk of LCF was out-weighted by more children in the placebo-group having ETF or PCR-adjusted treatment failure on day 7 or earlier indicating that paracetamol diminished the malaria symptoms in children and thereby also the need for being seen by a health professional or being admitted to hospital. Furthermore, both the cumulative ACPR-rates and the cumulative PCR-adjusted treatment failure rates on day 28 and day 35 were approximately identical in the two treatment groups. Therefore, the overall outcome of the treatment of malaria in children was not influenced negatively by adding paracetamol, though the children tended to experience more treatment failures at a later point when treated with paracetamol.

## Conclusion

In line with previous studies, hardly any differences in symptoms were found during treatment with paracetamol as compared to treatment with placebo. However, fewer (2 children vs. 6 children) children were admitted to hospital due to convulsions and high fever in the paracetamol group, though the finding was not statistically significant. Treatment of children suffering from uncomplicated malaria with paracetamol in the doses recommended did not influence the overall cure-rate, however the clinical beneficial effects are doubtful.

## Conclict of interest

The authors declare that they have no competing interests.

## Authors' contributions

PK participated in the design of the study, performed the statistical analyses and drafted the manuscript. JU performed the PCR analyses and interpreted the results, participated in preparing the manuscript. AR participated in designing the study and the daily supervision, participated in preparing the manuscript. LR participated in designing the study, participated in preparing the manuscript.All authors read and approved the final manuscript.
